# A Case of Urinary Sepsis Secondary to *Streptococcus sanguinis*

**DOI:** 10.1155/2019/7478607

**Published:** 2019-11-26

**Authors:** Ashley Reuter, Ashley Heyman, Benjamin Stockton, David Kraklau, Michael S. Wang

**Affiliations:** ^1^Spectrum Health Lakeland, Department of Medicine—St. Joseph, Osteopathic Medical Specialties, Michigan State University College of Osteopathic Medicine, East Lansing, MI, USA; ^2^Spectrum Health Lakeland, Department of Surgery—St. Joseph, Osteopathic Surgical Specialties, Michigan State University College of Osteopathic Medicine, East Lansing, MI, USA

## Abstract

We report a case of a 71-year-old male with a history of BPH who presented with flank pain, fever, chills, abdominal pain, and nausea. He had a dental cleaning 1 month prior to admission and flosses daily. Laboratory data revealed both urine and blood cultures to be positive for *streptococcus sanguinis.* Computed tomography revealed a 10 mm right ureteral stone, and an ultrasound demonstrated moderate right hydronephrosis. He underwent an ureteroscopy with stent placement. A transesophageal echocardiogram was negative for endocarditis. He completed 2 weeks of IV ceftriaxone and made a complete recovery.

## 1. Introduction

Sepsis, secondary to urinary tract infection (UTI), is most commonly caused by enteric bacteria and Gram-positive organisms (specifically enterococcus species) [1]. Eighty percent of cases are due to obstructive uropathy and forty-three percent are due to urolithiasis [1]. Risk factors for urinary sepsis include age _≥_65 years, diabetes mellitus, immune suppression, nosocomial urinary tract infection acquired in urology ward, and prior urological interventions [1]. Rare reports of *Streptococcus sanguinis* (*S. sanguinis*) have been reported, specifically associated with postureteral procedures [2]. To our knowledge, this is only the second case of *S. sanguinis* causing sepsis with UTI as a primary inciting event.

## 2. Case Presentation

**Figure 1:**
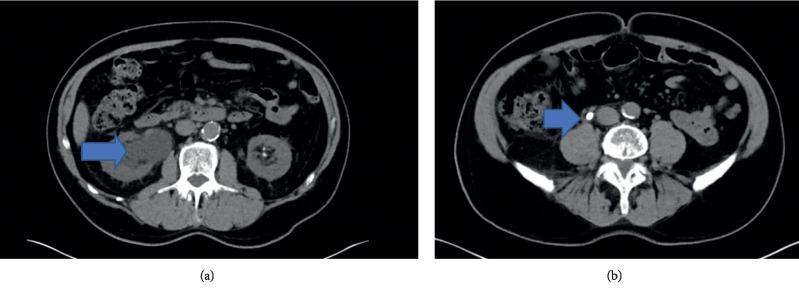
(a) Right kidney hydronephrosis and (b) right sided ureteral stone.

Case report: A 71-year-old male with a history of benign prostatic hyperplasia, nephrolithiasis, and coronary artery disease status/post coronary artery bypass graft was hospitalized for sepsis UTI and obstructive uropathy from nephrolithiasis. Three days prior to admission, he experienced right flank pain, followed by fever, chills, malaise, abdominal pain, and nausea. He took acetaminophen for pain. He denied dysuria, gross hematuria, prior urethral stricture, diarrhea, poor dentition, or alcohol use. He denied history of valve replacements or other indwelling devices/catheters. He flosses daily and follows with his dentist regularly, with teeth cleaning 1 month prior to presentation. He denied recent sexual activity. Physical exam was positive for fever (38.0 degree Celsius), tachycardia, and diaphoresis. Capillary refill was normal. Initial laboratory results showed mild leukocytosis, normocytic anemia, thrombocytopenia, acute kidney injury with creatinine 2.2, and estimated glomerular filtration rate 29. Urine showed microscopic hematuria and small leukocytes. Computed tomography abdomen/pelvis demonstrated moderate right hydronephrosis (Figure 1(a)) and a 10 mm right ureteral stone and multiple nonobstructing left renal calculi Figure 1(b).

The patient was treated with aggressive intravenous (IV) fluid hydration and IV Ceftriaxone. Urology was consulted, and a right ureteral stent was placed. Due to unresolving sepsis, antibiotics were broadened to IV Vancomycin and Piperacillin/Tazobactam. Blood and urine cultures resulted positive for *S. sanguinis*. Infectious disease was consulted for treatment of his streptococcus bacteremia. Additional workup was negative for infectious endocarditis including a transesophageal echocardiogram. Antibiotics were de-escalated to IV ceftriaxone 2 g every 24 hours. The patient completed a 2-week course of IV ceftriaxone. Follow-up blood cultures remained negative, and his renal function normalized. The right stent was later exchanged with lithotripsy of the right renal calculus.

## 3. Discussion

In terms of streptococcus species, it is well known that group B streptococcus is a colonizer of the urinary tract [3]. Previous laboratory testing has documented that viridians streptococci, including *Streptococcus sanguinis*, have been grown in urinary isolates, but it is unusual for isolates to be pathogenic [4, 5]. In addition, patients colonized or infected with urinary viridans streptococci have been overwhelmingly female [4, 5], which contrasts with our patient who is a male.

*S. sanguinis* is classified as a non-spore-forming, catalase-negative, chain-forming coccus and belongs to the non-beta-hemolytic, mitis group. The function of oral streptococci is to provide a favorable environment for later species to support mature oral biofilm, thus, providing protection for the oral cavity. In References [6, 7], *S. sanguinis* has been demonstrated to be associated with the initial stages of dental plaque formation [8]. The most common extraoral disease *S. sanguinis* plays a role in subacute infective endocarditis, most often from postdental procedures [6, 7]. Interestingly, our patient had a dental cleaning 1 month prior to hospitalization, but his oral cavity appeared normal. The time frame of *S. sanguinis* causing bacteremia after a dental procedure is unknown. The specific role of biofilm adherence and endocarditis has not been well established [7]. Other associated systemic diseases include meningitis (postinfection of the spinal cord lining or brain) and disseminated intravascular coagulation, causing activation of the coagulation cascade, forming small clots occluding blood flow to major organs and tissues [6].

To our knowledge, there is only one prior documented urinary tract infection caused by *S. sanguinis* [2]. This patient also presented with hydronephrosis, but there was no documented bacteremia [2]. The iliopsoas abscess was resolved by percutaneous drainage and medical treatment with ampicillin/sulbactam. There are few documented cases of iliopsoas abscess caused by urinary tract infections, which were primarily posturologic interventions and in association with ureteral stones [2].

Our patient was also found to have a right renal calculus and hydronephrosis which was likely the source of the *S. sanguinis* urinary sepsis. Penicillin susceptibility for *Streptococcus sanguinis* has been documented in the medical literature to be 60% [8]. Ceftriaxone and vancomycin susceptibility was shown to be 92% and 100% [8], respectively. The guidelines for infective endocarditis recommend penicillin G and ceftriaxone as 1st-line therapy for viridans streptoccus endocarditis [9]. Based on the susceptibility pattern and concern of potential dissemination, we elected to treat our patient with ceftriaxone for 14 days.

In this case, we report *S. sanguinis* causing urinary sepsis as a primary inciting event. Although *S. sanguinis* is an unusual cause of urinary tract pathology, treatment should be considered if the clinical picture is consistent with infection. Given the concern of dissemination, we recommend at least two weeks of antibiotics against *S. sanguinis* with repeated negative blood cultures prior to ureteral procedures.

## 4. Conclusions

*S. sanguinis* is a rare cause of sepsis due to UTI. There may be a correlation between nephrolithiasis and urinary tract infection caused by S*. sanguinis*. We recommend completing at least two weeks of *S. sanguinis*-directed antibiotics, followed by negative repeated blood and urine cultures prior to urologic procedures.
